# Role of the Cysteine in R3 Tau Peptide in Copper Binding and Reactivity

**DOI:** 10.3390/ijms231810726

**Published:** 2022-09-14

**Authors:** Chiara Bacchella, Silvia Gentili, Sara Ida Mozzi, Enrico Monzani, Luigi Casella, Matteo Tegoni, Simone Dell’Acqua

**Affiliations:** 1 Dipartimento di Chimica, Università di Pavia, Via Taramelli 12, 27100 Pavia, Italy; 2 Dipartimento di Scienze Chimiche, della Vita e della Sostenibilità Ambientale, Università di Parma, Parco Area delle Scienze 11/A, 43124 Parma, Italy

**Keywords:** tau protein, copper complexes, Alzheimer’s disease, oxidative stress, post-translational protein modification, cysteine oxidation

## Abstract

Tau is a widespread neuroprotein that regulates the cytoskeleton assembly. In some neurological disorders, known as tauopathies, tau is dissociated from the microtubule and forms insoluble neurofibrillary tangles. Tau comprises four pseudorepeats (R1–R4), containing one (R1, R2, R4) or two (R3) histidines, that potentially act as metal binding sites. Moreover, Cys291 and Cys322 in R2 and R3, respectively, might have an important role in protein aggregation, through possible disulfide bond formation, and/or affecting the binding and reactivity of redox-active metal ions, as copper. We, therefore, compare the interaction of copper with octadeca-R3-peptide (R3C) and with the mutant containing an alanine residue (R3A) to assess the role of thiol group. Spectrophotometric titrations allow to calculate the formation constant of the copper(I) complexes, showing a remarkable stronger interaction in the case of R3C (log *K*_f_ = 13.4 and 10.5 for copper(I)-R3C and copper(I)-R3A, respectively). We also evaluate the oxidative reactivity associated to these copper complexes in the presence of dopamine and ascorbate. Both R3A and R3C peptides increase the capability of copper to oxidize catechols, but copper-R3C displays a peculiar mechanism due to the presence of cysteine. HPLC-MS analysis shows that cysteine can form disulfide bonds and dopamine-Cys covalent adducts, with potential implication in tau aggregation process.

## 1. Introduction

Alzheimer’s disease is a progressive neurodegenerative disorder characterized by specific biological evidence, such as the presence of abnormal protein aggregates in the central nervous system. These deposits consist in both extracellular amyloid plaques, mainly composed by amyloid-beta fragments, and intracellular neurofibrillary tangles, generated by the unusual stacking between tau proteins [1,2,3].

**Figure 1:**
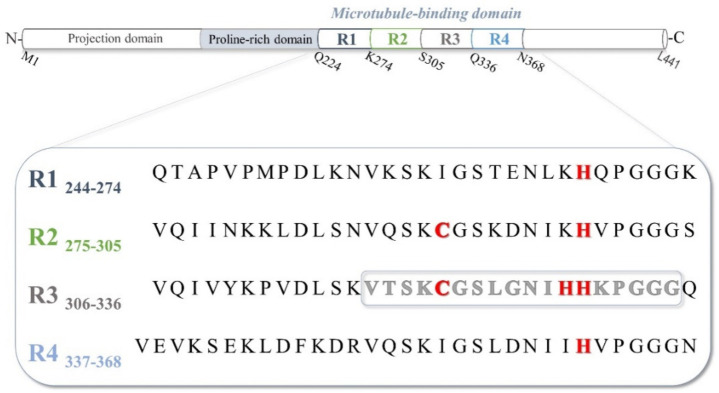
Schematic representation of the amino acid sequence of tau protein, highlighting the four pseudorepeats. The fragment used in this work is underlined in grey while the most relevant ligands for copper are shown in red.

Tau is a microtubule-associated protein mainly found in the axonal part of neurons. In adult human brain six tau isoforms are expressed that range from 352 to 441 residues [4]. They differ from each other by the presence or absence of one or two inserts (N1, N2) in the *N*-terminal part, and by the presence of either three repeats (R1, R3, R4) or four repeats (R1, R2, R3, and R4) in the *C*-terminal part. These pseudorepeats are formed by 31–32 amino acids including a highly homologous 18 residues sequence (Figure 1), and constitute the microtubule-binding domain. 

In pathological conditions observed in Alzheimer’s disease and other tauopathies, tau switches its conformation to abnormal filaments [5], which were defined as paired helical filaments since their discovery in 1963 [6]. This conformational change is associated with an abnormal phosphorylation, targeting the numerous serine, threonine and tyrosine residues present in tau [7,8,9,10]. Recent cryo-EM studies have allowed to analyze the structure of tau in pathological conditions, which is characterized by the formation of β-strand structure between residues 306 and 378, which matches with the portion of tau comprising R3 and R4 region [11,12].

However, the initial events inducing this aggregation mechanism have not been elucidated yet. Besides phosphorylation, which could be potentially treated by a series of inhibitors [13,14], a significant role in the aggregation process might be also played by two cysteine residues present in the tau sequence: Cys291 and Cys322 in R2 and R3 fragments, respectively. The role of these amino acids, for both physiological and pathological aspects, is still object of debate. In physiological conditions, it has been proposed that these residues are involved in the catalytic lysine acetylation [15] and microtubule binding [16,17]. Conversely, a crucial role for aggregation process of tau and, therefore, the spread of the pathology has been suggested for these residues. 

In vitro experiments have demonstrated that the oxidation of two Cys residues and the consequent formation of intermolecular disulfide bond induce a conformational change of tau structure making it more prone to aggregation [18,19,20,21,22], although other studies suggest that the formation of intramolecular disulfide bond leads to an aggregation-resistant form of tau [23,24,25,26]. A recent study has proposed that the two Cys residues, which are structurally different since only Cys322 is inserted into the core of the fibril [12], give different contribution to tau toxicity and dysfunction [27]. 

This evidence suggests that more attention should be given to factors that could result in a harmful oxidizing environment, such as oxidative stress and redox active metals. These two aspects are closely related to each other and are proposed to be involved in several neurodegenerative diseases [28,29].

In particular, copper, beside its role as a cofactor in several neuronal enzymes [30], serves as allosteric regulator of protein activity [31]. However, impairment of copper homeostasis has detrimental effect on cellular environment by contributing to an increase of production of reactive oxygen species (ROS) through Fenton or Haber–Weiss reaction [32]. The involvement of copper in Alzheimer’s disease is also associated to its important role in signaling at the synaptic cleft where, in pathological conditions, it modifies amyloid-β structure, aggregation, and toxicity [33]. Moreover, the role of copper in tau-related pathologies has been postulated and it is the subject of several studies [34,35]. In particular, copper increases the aggregation propensity of tau through both its capability to bind tau and produce ROS [36,37]. Copper binding occurs through histidine residues present in R1, R2, R3, and R4, [38,39,40,41,42,43] or at the *N*-terminal site [44,45]. A recent theoretical model of copper–R3 complex suggested that copper(II) ion induces α-helix folding of R3 peptide, even if the role of the cysteine residue has not been evaluated [46]. We have recently reported a detailed characterization of copper binding (in both oxidation states) to R1 and a “short” R3 fragment. A short R3 sequence (Ac-^323^GSLGNIHHKPGGG^335^-NH_2_) was used to study the role of the two histidines and to exclude the cysteine present in the full-length R3 peptide (Ac-^318^VTSKCGSLGNIHHKPGGG^335^-NH_2_) as an additional variable. That study showed that the His tandem present in the R3 fragment confers a stronger binding site for copper(II) when compared to single His residue in the R1 peptide (*K*_d_ = 150 nM for R1 and 71 nM for R3 at pH 7.4) [47]. Our previous analysis showed that the copper–R3 complex is able to promote the simultaneous oxidation of external substrates such as catechols, and in particular dopamine (DA), and the oxidation of the peptide backbone [47]. This reactivity might also cause the nucleophilic addition of quinone products to electrophilic amino acids as histidine or cysteine [48,49].

In the present study, we aim at clarifying how the presence of the cysteine in R3 affects the binding of copper and the reactivity of the corresponding complex. In particular, we compare the full-length octadeca-R3-peptide (R3C: Ac-^318^VTSKCGSLGNIHHKPGGG^335^-NH_2_) with the mutant containing an alanine residue in the 322 position (R3A: Ac-^318^VTSKAGSLGNIHHKPGGG^335^-NH_2_).

## 2. Results and Discussion

### 2.1. Protonation and Copper(II) Complexation Equilibria

The R3A peptide contains four basic amino acids (2 His, 2 Lys), and therefore R3A behaves as a tetraprotic acid when fully protonated (H_4_L^4+^). The proton dissociation constants determined by potentiometric titrations (reported in Table 1) are indeed fully consistent with the dissociation of the two histidine imidazolium groups (in the pH range 5.5–7.5) and of the two protonated lysine side chains (at pH > 9). At pH 7.4, the predominant form of R3A is H_2_L^2+^. 

### 2.2. Copper(II) Complexation Studies

The complexation of copper(II) with the tau fragment R3A was studied by potentiometric titrations in aqueous solution. Potentiometric data were complemented by visible absorption and circular dichroism characterization of Cu^2+^/peptide system at different pH values, to provide with information on the coordination environment of copper in the complex species. The same experiments could not be carried out for the R3C fragment since rapid oxidation of the peptide at the Cys residue was observed even at low pH in the presence of copper(II), as demonstrated by the marked drift in the e.m.f. values even in mild acidic pH conditions observed during attempted potentiometric studies.

#### Cu^2+^/R3A Complex Formation Equilibria

In the presence of copper(II), the R3A peptide forms six complex species, all with 1:1 copper/peptide stoichiometry and differing for their protonation states (Table 1). The analysis of the potentiometric curves suggests no formation of either 2:1 or 1:2 copper(II)/peptide species under our experimental conditions.

**Figure 2:**
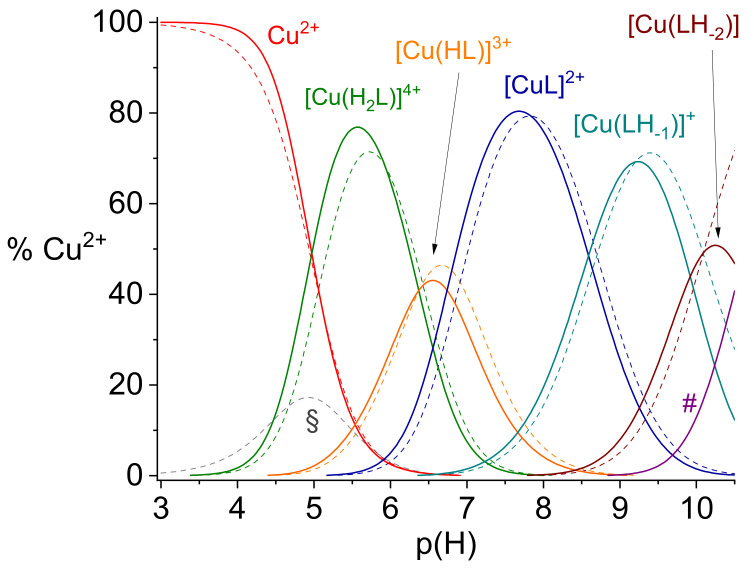
Straight lines: representative distribution diagram for the system Cu(II)/R3A (L = R3A). Cu/R3A = 1:2.1, [Cu] = 0.55 mM, *T* = 298.2 K, *I* = 0.1 M (KCl). Curve marked with # = [Cu(LH_−3_)]^−^. Dashed lines: representative distribution diagram for the system Cu(II) R3 (same conditions, see ref. [47]). The dashed curve indicated with § relates with the [Cu(H_2_L)]^4+^ of L = R3.

**Figure 3:**
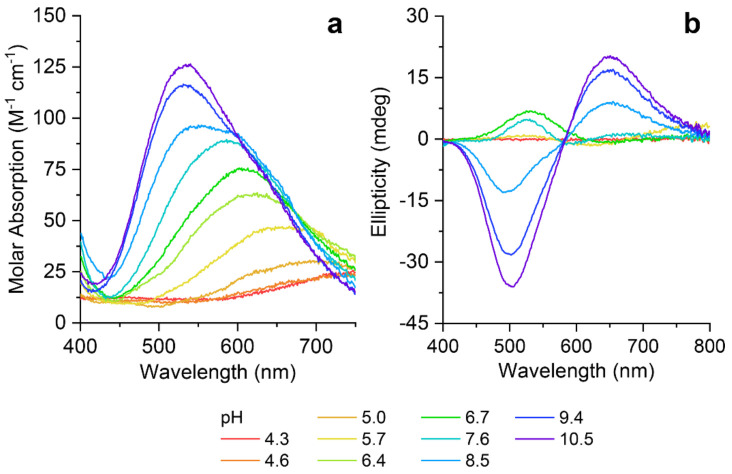
(**a**) Visible molar absorption spectra (absorbance/total [Cu]) of solutions of copper(II) and R3A at different pH (Cu^2+^/R3A = 1:2.1, [Cu] = 0.70 mM, *T* = 298.2 K, *I* = 0.1 M in KCl). (**b**) Visible CD spectra of solutions of copper(II) and R3A (Cu^2+^/R3A = 1:2.1, [Cu] = 0.52 mM, *T* = 298.2 K, *I* = 0.1 M in KCl).

**Scheme 1:**
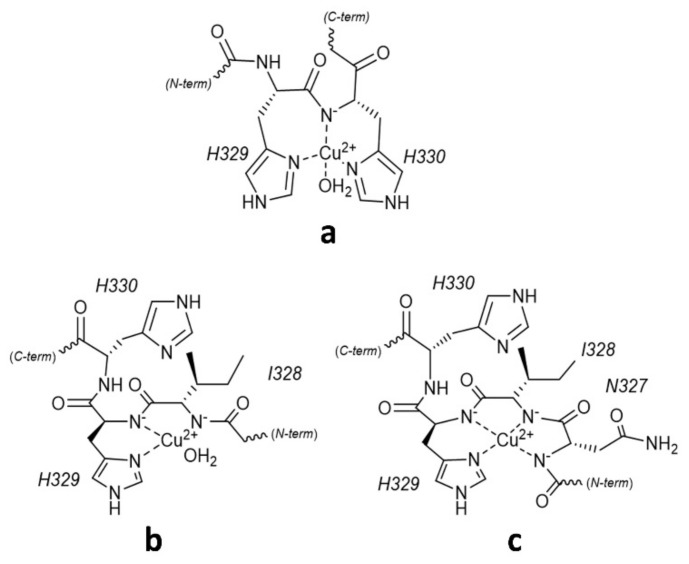
Schematic representations of the copper(II) coordination in: (**a**) [Cu(HL)]^3+^, (**b**) [CuL]^2+^, and (**c**) [Cu(LH_−1_)]^+^ of the R3A peptide (R3A = L).

The distribution diagram is reported in Figure 2 (straight lines) overlapped with that of R3 (dashed lines, see ref. [47]). Although the protonation states of the two ligands differ of one proton (i.e., one extra acidic group in R3A), it is evident that the distribution diagrams of the two peptides are almost superimposable, and the interpretation of the potentiometric and spectral data is fully consistent with this observation (see below). The predominant species below pH 6.2 is [Cu(H_2_L)]^4+^, which starts forming at pH 4 and reaches ca. 80% of total copper at pH 5.6. The visible absorption spectrum recorded at pH 5.7 (Figure 3a) shows a broad band at ca. 660 nm, that is well accounted for a (N_im_, N^−^, 2x O_w_) donor set as calculated based on the Billo’s average environment theory (calc. λ_max_ 661 nm). By increasing the pH, the species [CuHL]^3+^ becomes predominant, and at pH 6.6 it accounts for ca. 40% total copper. The observed λ_max_ at ca. 610 nm at pH 6.7 (Figure 3a) suggests a (2× N_im_, N^−^, O_w_) coordination mode for copper(II) in this species (calc. λ_max_ 608 nm, Scheme 1a). The absorption band at pH 6.7 is broad toward lower wavelength for the spectral contribution of the [CuL]^2+^ species which is present at ca. 30% total copper, and which absorbs at 580 nm (see below). 

At pH 7.4 the speciation is dominated by [CuL]^2+^ (ca. 75%) which reaches a maximum of ca. 80% total copper at pH 7.6. For this species we predict a (N_im_, 2x N^−^, O_w_) coordination for copper(II), which is consistent with the experimental λ_max_ of 584 nm at pH 7.6, indeed coincident with the value expected for this copper(II) coordination environment. Likely the imidazole coordinated to the equatorial site on copper(II) is that of His329, while the peptide nitrogen atoms are those of H329 and I328. This coordination mode also allows the imidazole of His330 to possibly axially coordinate to copper(II) (Scheme 1b). 

Moving from pH 7.6 toward higher pH values, a blue shift of the absorption band was observed. This clearly indicates the formation of other species, which is discussed in Supporting Information.

Moreover, Figure S1 shows that the increase of absorbance at 610 nm starts at pH ca. 5, and is in agreement with the curve of formation of the [CuHL]^3+^ species (Figure 2). Table S1 also reports the main spectroscopic absorption features for Cu(II)/R3A system.

### 2.3. Cu^+^/R3 and R3A Complex Formation Equilibria

The stability of the complexes of Cu(I) with peptides R3C and R3A was determined by spectrophotometric titrations of solutions containing [Cu^I^(CH_3_CN)_4_]^+^ and sodium bicinchoninate (BCA) or ferrozine (Fz) for the two peptides, respectively. While the use of Fz resulted appropriate for R3A, the use of BCA in the case of R3C was necessary as the adduct of Cu(I) with this peptide resulted in remarkable stability (see below).

**Figure 4:**
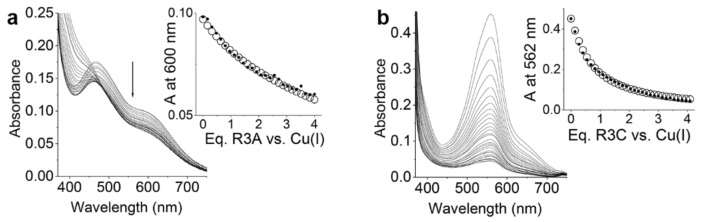
(**a**) Spectral data set for the titration of a solution of [Cu(CH_3_CN)_4_]BF_4_ and sodium bicinchoninate (BCA) (Cu/BCA = 1:2.5, [Cu] = 55 μM) with increasing concentration of R3C, up to 4.2 eq. (**b**) Spectral data set for the titration of a solution of [Cu(CH_3_CN)_4_]BF_4_ and ferrozine (Fz) (Cu/Fz = 1:3.1, [Cu] = 55 μM) with increasing concentration of R3A, up to 4.0 eq. For both datasets, C_ascorbate_ = 10 mM, 100 mM aqueous HEPES buffer solution, pH 7.4.

Figure 4 reports the spectrophotometric titrations of solutions of Cu(I) with BCA or Fz at pH 7.4 in HEPES buffer. Both datasets show a decrease in the intensity of the visible absorption band of the Cu(I)/indicator adducts (Figure 4) upon addition of the peptides. This demonstrates to the naked eye the competition between the indicators and the peptides in Cu(I) binding following the equilibrium: Cu(I)(indicator)_2_ + peptide ⇄ Cu(I)(peptide) + 2 indicator. These titrations were preceded by spectrophotometric titrations of solutions of the indicators with Cu(I) to determine the molar absorbance of the Cu(I)/indicator complexes under our experimental conditions. The values of the formation constants *K*_f_ of the Cu(I)/peptide complexes were calculated from the dataset in Figure 4 using the speciation reported in the literature [50,51]. Results of data treatment suggest that both R3C and R3A form 1:1 complexes with Cu(I) of log *K*_f_ = 13.4(1) and 10.5(1) corresponding to *K*_d_ of 0.40 and 32 pM for the two peptides, respectively. As for the stability of the complex of Cu(I) with R3A, it results within the same order of magnitude as that reported for the analogous shorter R3 fragment (log *K*_f_ = 10.1(1)) [47]. Conversely, the formation constant of the Cu(I)/R3C complex is 3 orders of magnitude larger compared to that of R3A suggesting that the soft Cys residue is coordinated to copper(I) at pH 7.4. 

### 2.4. Oxidation of Dopamine and 4-Methylcatechol by Copper–R3C and Copper–R3A Complexes

As for our previous analysis of the affinity of copper for R1 and the short R3 fragment [47], it is important to evaluate the reactivity associated to copper–tau complexes in order to clarify their possible role in the oxidation of external substrates, such as catecholamine neurotransmitters, and in the peptide self-modification, i.e., the oxidative modifications of the bound peptide. Dopamine (DA) is the most relevant substrate for this reactivity since it is present at relatively high concentration in the brain, particularly in the *substantia nigra*, and impairment in its metabolism and homeostasis can contribute to neurodegenerative diseases [52,53].

**Scheme 2:**
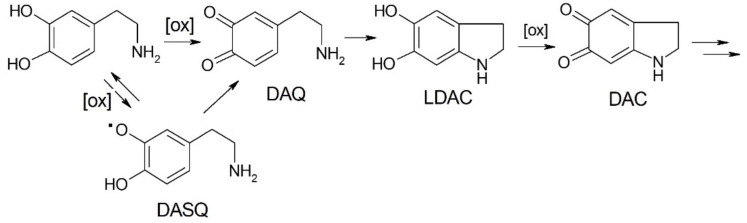
Initial steps of DA oxidation promoted by oxidizing species, generically indicated as [ox]. DAQ: dopamine quinone; DASQ: dopamine semiquinone; LDAC: leukodopaminochrome; DAC: dopaminochrome. DAC undergoes further oxidation with formation of insoluble conjugates.

The initial steps of DA oxidation are illustrated in Scheme 2. Initial formation of dopamine quinone (DAQ) is followed by an intramolecular cyclization to leukodopaminochrome (LDAC), an unstable intermediate easily converted to dopaminochrome (DAC) [53]. This product is stable within approximately one-hour reaction time, before the formation of insoluble melanic products, allowing the analysis by UV-visible spectroscopy through the development of its absorption band at 475 nm [54].

**Figure 5:**
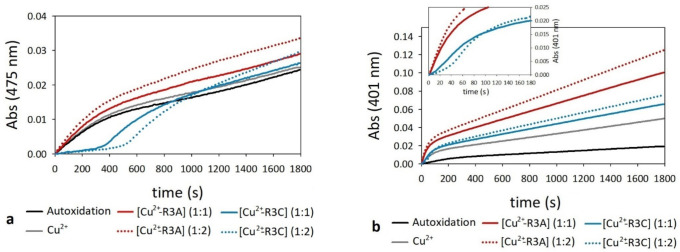
Kinetic profiles of DA (panel **a**, 3 mM) and MC (panel **b**, 3 mM) oxidation with time in 50 mM HEPES buffer at pH 7.4 and 25 °C promoted by atmospheric dioxygen (autoxidation, black trace), copper(II) alone (25 µM, grey), and in the presence of R3A (red traces) or R3C peptides (light blue traces) (25 µM, solid and 50 µM, dotted). Panel b also shows an enlargement of the first 180 s to better evidence the initial sigmoidal trend of the kinetics.

As shown by the black trace in Figure 5a, DA (3 mM) reacts spontaneously with dioxygen in HEPES buffer at pH 7.4 and 25 °C and the presence of copper(II) (25 µM) slightly increases the reaction rate (Figure 5a—grey trace). The addition of 1:1 or 2:1 R3A or R3C peptide to copper ratios displays a highly different behavior. The formation of Cu–R3A complex enhances the oxidation promoted by copper(II) (Figure 5a—red traces); this result is in agreement with our previous study performed with the shorter R3 peptide (Ac-^323^GSLGNIHHKPGGG^335^-NH_2_) lacking the Cys residue but possessing the His-His tandem [47]. Conversely, the presence of R3C induces a different reactivity that is distinguished by a kinetic trace exhibiting an initial lag phase with a much slower DAC formation, followed by a delayed start of product formation. The length of the lag phase increases by increasing the peptide concentration, as shown by the solid and dotted light blue traces.

Before further investigation on the nature of this lag phase (see below), we also investigated the oxidation of more reactive 4-methylcatechol (MC), often used as model substrate of DA, due its lower catechol/semiquinone redox potential and simpler pattern of oxidation products compared to DA [55]. The formation of 4-methylquinone at 401 nm proceeds with a biphasic behavior which is accentuated by the catalysis promoted by copper(II) (Figure 5b—grey trace) compared to MC autoxidation (Figure 5b—black trace). The presence of R3A peptide increases the rate of both steps, in agreement with the previously observed behavior with the short R3 peptide [47]. The addition of 1 or 2 equivalents of R3C induces an increase in the rate of the second phase of the reaction which is lower compared to that of copper–R3A complex. In the first phase, within the first 100 s, the kinetic oxidation trace shows a weak sigmoidal trend that resembles the same effect observed with DA oxidation by copper–R3C complex but with a much shorter lag phase in this case (see enlargement in Figure 5, panel b).

For copper–R3A complex, we can assume that this reaction proceeds with the same mechanism proposed for copper–R1 and copper–R3 complexes [47], and analogue to that of copper-Aβ [56], copper-α-synuclein [55,57], and copper-prion [58] peptide complexes. The mechanism involves the following reactions:

1. Cu^2+^ + peptide ⇄ [Cu^2+^-peptide]
2. [Cu^2+^-peptide] + catechol → [Cu^+^-peptide] + semiquinone^•+^
3. [Cu^+^-peptide] + catechol ⇄ [Cu^+^-peptide-catechol]
4. [Cu^+^-peptide-catechol] + O_2_ → [Cu-peptide-catechol-O_2_]
5. [Cu-peptide-catechol-O_2_] →→ [Cu^2+^-peptide] + quinone
6. 2 semiquinone^•+^ → catechol + quinone

After the copper–peptide complex formation (reaction 1), the reaction proceeds with the reduction of copper(II) to copper(I) by the catechol and formation of a semiquinone intermediate (reaction 2); this reaction, followed by reaction 6, is responsible for the first fast reaction observed in the biphasic trend, particularly evident for MC substrate. Reaction 3 is a pre-equilibrium that allows the catechol binding to the copper(I)–peptide complex, followed by oxygen binding to generate the ternary complex indicated as [Cu-peptide-catechol-O_2_] (reaction 4), which is the rate-determining step of the catalytic process. The charge of the species involved in this intermediate is omitted because the characterization of this intermediate is made difficult by its transient nature, especially at room temperature. 

**Figure 6:**
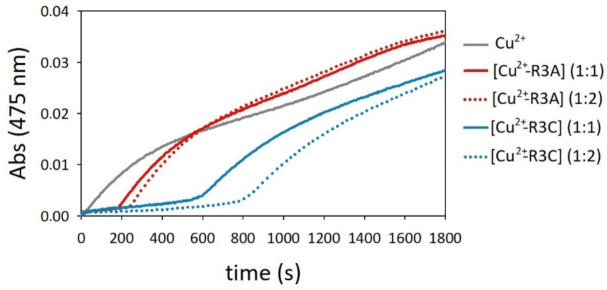
Kinetic profiles of DA (3 mM) oxidation with time in 50 mM HEPES buffer at pH 7.4 and 25 °C promoted by copper(I) alone (25 µM, grey trace) and in the presence of R3A (red traces) or R3C peptides (light blue traces) (25 µM, solid and 50 µM, dotted). Tetrakis(acetonitrile)copper(I) hexafluorophosphate was previously dissolved in pure acetonitrile and purged with argon.

Since the formation of the copper(I) intermediates plays a crucial role for the oxidative catechol catalysis, a further experiment was performed in which DA oxidation is started by adding copper(I) to the air-saturated reaction mixture. Figure 6 shows that free copper(I) (grey trace) reacts rapidly with dioxygen giving rise to an instantaneous development of oxidative products, whereas the lag phase observed with both R3A and R3C peptides indicates a stabilization of copper–peptide complex in the reduced form. The longer lag phase for copper(I)-R3C might depend on the different copper(I) environment and on the high binding affinity for the metal in this oxidation state (log *K*_f_ for Cu(I), see above). It should be noted that during the lag phase, the reaction is much slower but not completely quenched. On the other hand, even if the length of the lag phase is different, we can observe that the slopes of the kinetic traces in correspondence of the substrate oxidation are comparable with both R3A (red traces) and R3C peptides (light blue traces). This result indicates that even if copper(I) coordination is different, as indicated by affinity constant determination reported above, the “catalytically competent” site is similar in the two cases, with the cysteine residue not involved in metal coordination sphere.

**Figure 7:**
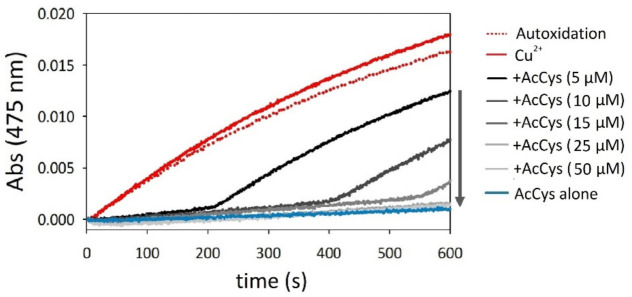
Kinetic profiles of DA (3 mM) oxidation with time in 50 mM HEPES buffer at pH 7.4 and 25 °C catalyzed by atmospheric dioxygen (autoxidation, dotted red trace), copper(II) alone (25 μΜ, solid red), and in the presence of *N*-acetylcysteine (5, 10, 15, 25, 50 μM, black to gray). Light blue trace is obtained only in the presence of *N*-acetylcysteine (25 μΜ) without the metal.

To identify the role of cysteine in the reaction mechanism, a control experiment was performed in absence of R3C but in the presence of free copper, DA, and increasing amount of *N*-acetylcysteine (Figure 7). The increase in concentration of *N*-acetylcysteine corresponds to an increase in the lag phase duration, confirming that the absence of DAC band development is correlated to the concentration of free cysteine in solution, not necessarily being part of the peptide backbone. At the opposite, the presence of the “oxidized cysteine” (*N*-acetylcystine) does not affect the rate of the copper-mediated DA oxidation (Figure S2).

**Figure 8:**
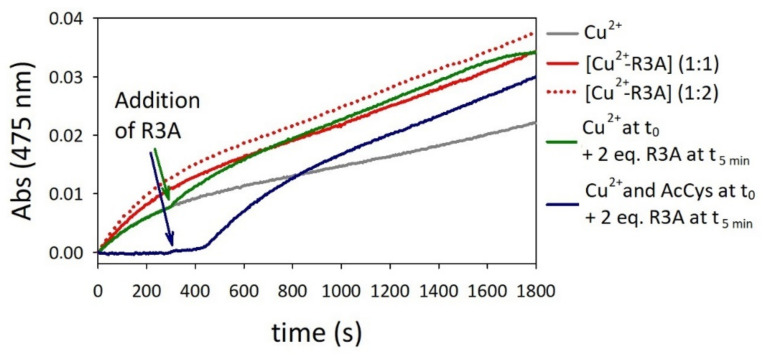
Kinetic profiles of DA (3 mM) oxidation with time in 50 mM HEPES buffer at pH 7.4 and 25 °C catalyzed by: (i) copper(II) alone (25 μM, grey trace); (ii) copper(II) (25 μM) and 1 equiv. (25 μΜ, solid red) and 2 equiv. R3A (50 μΜ, dotted red); (iii) copper(II) alone (25 μM) and further addition of 2 equiv. R3A after 5 min of reaction (solid green); (iv) in the same conditions of point (iii) but with the addition of *N*-acetylcysteine (5 μM, solid blue).

The capability of cysteine to “quench” the DAC formation is confirmed by an experiment in which the R3A peptide, which enhances the oxidation promoted by copper (see above, Figure 5a) is added to a reaction mixture containing free copper (Figure 8—solid green trace) or to a solution that contains a sub-stoichiometric amount of *N*-acetylcysteine (Figure 8—solid blue trace). Again, DAC formation is prevented until *N*-acetylcysteine is in solution.

Having established that the lag phase observed with R3C is due to the presence of cysteine residue, different hypothesis on the reaction mechanism can be proposed. The first hypothesis is that the Cys residue of the peptide could be competitively oxidized by copper(II), or by the reactive [Cu-peptide-O_2_] species (reactions 7–9), preventing copper-mediated catechol oxidation.

7. [Cu^2+^-peptide] + R3C → [Cu^+^-peptide] + R3C-S^•^ + H^+^
8. [Cu^+^-peptide] + O_2_ → [Cu-peptide-O_2_]
9. [Cu-peptide-O_2_] + R3C → [Cu^2+^-peptide] + R3C-S^•^ + H^+^

However, if this hypothesis were correct, the autoxidation of DA (independent by copper catalysis) would be still detectable by the formation of DAC bands as in the black trace of Figure 5a. Contrarily, the lag phase observed with R3C leads to discarding this hypothesis as the only responsible one for the reaction profile.

**Scheme 3:**
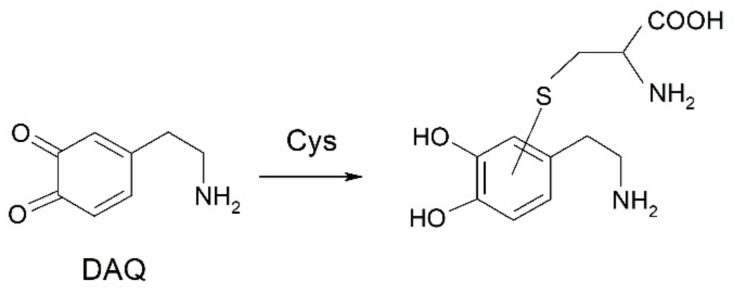
Michael type condensation reaction between DAQ species and nucleophilic amino acid side chains of Cys.

A second hypothesis is that cysteine reacts with the intermediate oxidation products of DA described in Scheme 2, preventing the formation of the DAC chromophore. Two different mechanisms are possible: the first is a one-electron radical process in which semiquinone intermediate reacts with cysteine (reactions 10–12), whereas the second is a Michael type condensation between cysteine and dopaminoquinone (Scheme 3). This reaction can also occur with histidine or lysine side chains, but the thiol group of cysteine is the most reactive sidechain functionalization in the nucleophilic addition to quinone. This reaction is indeed 10^6^-fold faster for cysteine comparing to histidine [59].

10. DA →→ →DASQ^•^
11. Cys-SH + DASQ^•^ → Cys-S^•^ + DA
12. 2 Cys-S^•^ → Cys-S-S-Cys

These reactions have relevant biological implications for proteins and peptides that contain cysteine residues, since both disulfide bond and DA-Cys adduct formation represent post-translational modifications that may alter the properties of proteins [49]. The formation of the disulfide bond might have a dual role in neurodegenerative disorders: on the one side, intramolecular binding could stabilize protein folding in the monomeric state, on the other side intermolecular interactions might alter the aggregation process [60]. The formation of protein-DA adducts (also known as “dopamination” process) is a hallmark of increased oxidative stress, which has particular relevance for proteins related to Parkinson’s disease (i.e., α-synuclein, DJ-1, glucocerebrosidase, parkin etc.) [53,61].

In our system, both hypotheses agree with the absence of DAC chromophore as long as reducing cysteine is available in solution. To elucidate which mechanism is active, it is necessary to identify the type of modification undergone by the peptide by HPLC-MS, as discussed in the following paragraph.

**Figure 9:**
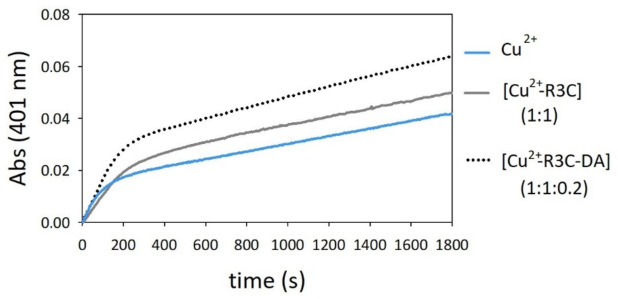
Kinetic profiles of MC (3 mM) oxidation with time in 50 mM HEPES buffer at pH 7.4 and 25 °C promoted by copper(II) alone (25 µM, light blue) and in the presence of R3C peptide (25 µM, solid grey). Dotted black trace was obtained by adding the pre-incubated (2 min) complex [copper (25 µM)-R3C (25 µM)-dopamine (5 µM)] to the substrate (3 mM).

Additionally, we compared the catecholase activity of Cu–R3C complex with that of a solution obtained by pre-incubating the metal–peptide complex with a micromolar amount of dopamine (see Figure 9), to evaluate if the modified peptide (following the two mechanisms proposed) displays an additional contribution to the oxidative reactivity. The resulting products exhibit a slightly higher catalytic efficiency when compared to the initial complex, mainly in the first stage of the reaction (within first 200 s of the reaction). We can therefore conclude that oxidative modifications (formation of DAQ-condensed R3C species or (R3)Cys-S-S-Cys(R3) dimer) induce an increase in the copper-mediated oxidation rates. 

### 2.5. Competitive Endogenous R3C and R3A Peptide Oxidation

**Figure 10:**
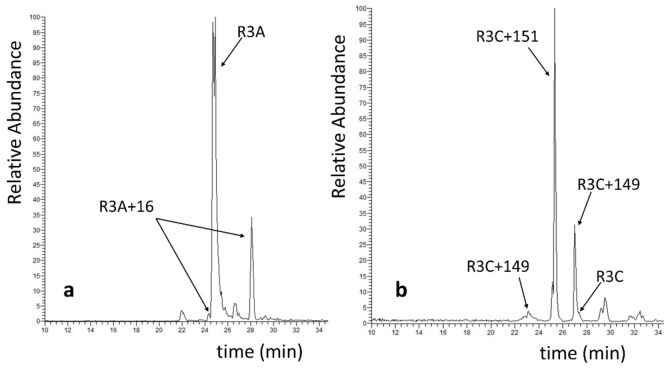
HPLC-MS elution profiles of (**a**) R3A and (**b**) R3C peptide (25 µM) in HEPES buffer (50 mM) pH 7.4 in the presence of copper(II) (25 µM) and DA (3 mM) after (**a**) 60 and (**b**) 10 min reaction time. The modifications shown in the chromatograms are the insertion of a single *O*-atom (+16) or the dopamination as covalent addition of DA (+151) and +DAQ (+149). Minor peaks at 22 and 26.5 min in panel (**a**) and peaks at 29 and 33 min in panel (**b**) were ascribable to fragments due to peptide hydrolysis; the mass and the nature of modification were not univocally identified.

The identification of peptide modifications upon copper/R3C/DA reaction is extremely relevant to determine which is the main mechanism involved. Following the examples of our previous studies, [47,55,56,58,62] we have performed a HPLC-MS analysis of the reaction mixture to identify and quantify the oxidative modification underwent by the peptide backbone. The R3A fragment undergoes a limited pattern of modifications in the presence of copper(II) and DA (Figure 10a), whereas in the presence of the most reactive MC, the chromatogram shows a larger number of modifications (Figure S3). Table 2 and Table S2 report the variation over time of the percent modification of the native peptide. Table 2 shows that in the presence of DA the most abundant modification undergone by R3A peptide is an oxygen atom insertion (R3A + 16).

In the presence of MC (Table S2), the percentage of unmodified R3A peptide drastically diminishes over time, and the major modification is still oxygen atom insertion, followed by a double oxygen atom insertion (R3A + 32). Moreover, a consistent amount of double modification consisting of 4-methylquinone (MQ) addition to one His residue (or Lys) (+120) and an *O*-atom insertion into His (+16), yielding a total mass increment of 136 and a double modification consisting of MC addition to one His/Lys residue (+122) and an *O*-atom insertion into His (+16), yielding a total mass increment of 138 is observed.

As expected, the R3C peptide displays a higher tendency to undergo oxidative modifications when compared to the R3A peptide. Indeed, HPLC/MS analysis in the presence of copper and MC shows that R3C is almost completely modified after 10 min time reaction (Figure S4). Even if the reaction is very fast, it is interesting to analyze the pattern of modification. Table S3 shows that the main modifications consist in MC or MQ addition to the peptide, with mass increment of +122/+120, respectively. The area of these two modified peptides, taken together, reaches 90% after 10 min. MS-MS fragmentation analysis does not allow to distinguish uniquely the residue involved in the adduct formation but the higher reactivity suggests cysteine as the residue covalently modified rather than histidine or lysine residues.

The kinetic study described above suggests also that a radical mechanism promoting disulfide bond formation is also possible. In fact, the HPLC-MS analysis shows the presence of the R3Cys-CysR3 dimer, but the extent of this modification is very low (1%). Anyway, the percentage of dimer formation could be underestimated since this modification may occur together with side chain oxidation and/or adduct formation; this complex pattern cannot be easily identified by the Bioworks software.

The same analysis was also performed with DA (Figure 10b). The presence of this less reactive substrate induces a slower modification of the peptide, even if R3C is almost completely modified after 30 min (Table 3). Again, the main modification observed is the formation of Cys-DA or Cys-DAQ adducts (70% and 12% after 30 min, respectively); the amount of dimer is slightly larger when compared to the experiment with MC (2% after 5 min and 4% after 30 min).

Finally, the presence of free *N*-acetyl-Cys (Figure S5) protects R3C, since the percentage of unmodified peptide increases from 5 to 42% after 10 min (Table 4). Moreover, the number of products due to Michael addition is lower, whereas the products from radical reaction (peptide–peptide dimer and peptide-AcCys adduct) are more abundant (2% and 9%, respectively). These results indicate that free cysteine is more reactive when compared to that inserted in the peptide backbone.

In conclusion, the HPLC-MS analysis allowed to identify the type and the extent of the peptide modifications in oxidative conditions. This analysis is particularly relevant in the presence of copper–R3C complex because the two reaction mechanisms described in the kinetic analysis would lead to a different modification pattern. In particular, the HPLC-MS analysis suggests that the Michael type condensation between cysteine and dopaminoquinone is the predominant mechanism, even if a concomitant one-electron radical process, which leads to disulfide bond formation, is also active.

### 2.6. Oxidation of Ascorbate by Copper–R3C and Copper–R3A Complexes

Both kinetic and HPLC-MS analysis indicate that the oxidative reactivity of copper-R3C and copper-R3A is different due to the presence of a cysteine residue in R3C. However, the mechanism observed is deeply affected by the presence of DA (or MC). In particular, the kinetic analysis has shown that a direct oxidation of Cys by copper(II) is not the prominent reaction when DA is present in solution. 

**Figure 11:**
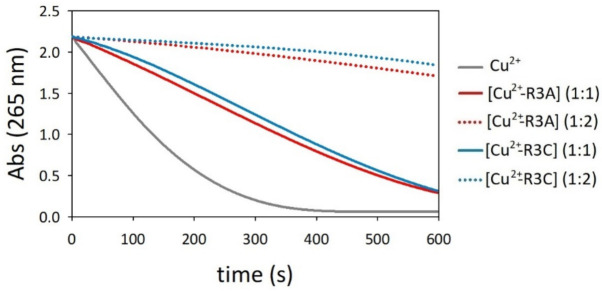
Ascorbate (0.15 mM) oxidation with time in 50 mM HEPES buffer at pH 7.4 and 25 °C promoted by copper(II) alone (25 µM, grey trace) and in the presence of R3A (red traces) or R3C (light blue traces) peptides (25 µM, solid and 50 µM, dotted).

It is therefore interesting to evaluate the capability of copper–peptide complexes in ROS production in the presence of another reducing agent that has high biological relevance as ascorbate. This vitamin is present at high concentrations intra- and extracellularly in the brain, and its oxidation by metal ions like copper and iron yields ROS formation [63]. The consumption of ascorbate promoted by copper(II) was monitored by the decrease in the absorption at 265 nm (Figure 11—grey trace). The presence of both R3C (Figure 11—light blue traces) and R3A (Figure 11—red traces) diminishes the rate of ascorbate oxidation; this behavior is in agreement with that observed with other copper complexes of neuronal peptides, such as those with *N*-terminal peptide fragments of β-amyloid and α-synuclein [64]. The slightly steeper slope for copper(II)–R3A compared to copper(II)–R3C complexes indicates that the oxidation of cysteine residue in R3C peptide competes with ascorbate oxidation. 

Similar results were reported in a previous study, where the copper-mediated ascorbate oxidation was followed at prolonged incubation time (24 h) [65]. In this analysis, all R1, R2, R3, and R4 peptides reduce the ability of copper to oxidize ascorbate; however, R3 peptide is slightly less efficient compared to the other peptides.

## 3. Materials and Methods

### 3.1. Materials and Instrumentation

All reagents for the peptide synthesis, including protected amino acids and rink amide resin, were purchased from Novabiochem, while other chemical compounds were reagent grade from Sigma-Aldrich. Purification of tau peptides was performed on a Shimadzu HPLC instrument equipped with two LC-20AD pumps and a SPD-M20A diode array detector, using a Phenomenex Jupiter 4µ Proteo semipreparative column (4 μm, 250 × 10 mm). Mass analysis and LC-MS/MS chromatograms were acquired with a LCQ ADV MAX ion-trap mass spectrometer, with an ESI ion source. The instrument works in automated LC-MS/MS mode through a surveyor HPLC system (Thermo Finnigan, San Jose, CA, USA) equipped with a Phenomenex Jupiter 4µ Proteo column (4 µm, 150 × 2.0 mm). In order to identify the oxidative modifications of peptide fragments, Bioworks 3.1 and Xcalibur 2.0.7 SP1 software were used (Thermo Finnigan, San Jose, CA, USA). UV-visible absorption spectra were collected with a Thermo Evolution 260 Bio spectrophotometer, provided with a Peltier thermostat. Circular dichroism spectra were acquired with a Jasco J715 spectropolarimeter, equipped with a Peltier thermostat. UV-visible kinetic profiles were obtained through an Agilent 8453 diode array spectrophotometer, equipped with a thermostated, magnetically stirred optical cell.

### 3.2. Peptide Synthesis

R3 fragment (R3C, Ac-^318^VTSKCGSLGNIHHKPGGG^335^-NH_2_) and its mutant with the substitution of Cys by Ala at position 322 (R3A, Ac-^318^VTSKAGSLGNIHHKPGGG^335^-NH_2_) were synthesized using the standard fluorenyl methoxycarbonyl (Fmoc) solid-phase synthesis in DMF [66,67]. The polymeric support chosen for the peptide growth was the Rink-amide resin MBHA (substitution 0.58 mmol/g), which releases, after cleavage, the peptide in the amidated form at the *C*-terminus. The deprotection of Fmoc groups from the resin and from each amino acid was achieved with 20% (*v*/*v*) piperidine in DMF, repeating the reaction twice, for 3 and 7 min. Coupling reaction of each amino acid (2 mol equiv. vs. resin sites) was performed in the presence of 2 equiv. of *N*-hydroxybenzotriazole (HOBt), 2 equiv. of benzotriazol-1-yloxytripyrrolidinophosphonium hexafluorophosphate (PyBOP), and ∼2 equiv. of *N*,*N*-diisopropyl ethylamine (DIEA). The reaction proceeds for about 45 min at room temperature, and it was repeated twice. Capping of the unreacted chains was done by using a solution of 4.7% (*v*/*v*) acetic anhydride and 4% (*v*/*v*) of pyridine in DMF. At the end of the synthesis, the resin was washed with DMF, dichloromethane, isopropanol, and diethyl ether; the cleavage of R3A peptide from the resin and the deprotection of the side chains of its amino acids were performed with a solution of 95% trifluoroacetic acid (TFA, 25 mL for 1 g of resin), triisopropyl silane (2.5%), and water (2.5%). For the sequence containing the Cys residue, the cleavage mixture was modified by using triisopropyl silane (1%) and by further addition of 1,2-ethanedithiol (2.5%). The mixtures were stirred for about 3 h; cold diethyl ether was used to allow the precipitation of the products and then recovered through filtration. Crude peptides dissolved in water were purified by HPLC, using a 0–100% linear gradient of 0.1% TFA in water to 0.1% TFA in CH_3_CN over 26 min (flow rate of 3.2 mL/min, loop 2 mL), as eluent. Both purified peptides were lyophilized and stored at −20 °C and, for R3C fragment, several vacuum/argon cycles were performed to prevent the oxidation of the Cys thiol group. The identity of the peptides was checked by electrospray ionization mass spectrometry (Thermo-Finnigan). ESI-MS data (direct injection, MeOH, positive-ion mode, capillary temperature 200 °C): *m*/*z* 1759 (R3AH)^+^; 880 (R3AH_2_)^2+^; 587 (R3AH_3_)^3+^; 440.5 (R3AH_4_)^4+^; 1791 (R3CH)^+^; 896 (R3CH_2_)^2+^; 597.7 (R3CH_3_)^3+^; 448.5 (R3CH_4_)^4+^. 

### 3.3. Complex Formation Equilibria Studies

The stock solution of R3A was prepared at 4.5 mM concentration in high purity, double distilled H_2_O with the addition of 1 equiv. of HCl (pH ca. 2.3). The titer of the solution was checked potentiometrically. The stock solutions of R3C were prepared at 1.3–1.9 mM concentration in high-purity D_2_O and the peptide concentration determined by NMR using an external standard. Both solutions were divided into 100 μL aliquots and stored at −20 °C. The ferrozine sodium salt (Fz) and bicinchoninic acid sodium salt (BCA) stock solutions were prepared by weight at 1.0–1.2 mM concentrations in HEPES buffer solution (100 mM, pH 7.4). Copper(II) chloride stock solutions (14.7 mM) were prepared by weight in doubly distilled water and their titer determined by complexometric titrations with standard EDTA solutions. Solutions of [Cu(CH_3_CN)_4_]BF_4_ in acetonitrile (1.15 mM) were prepared by weight and their titers were checked by addition of an excess of BCA (Cu(I) 100 µM in HEPES 0.1 M, NaAsc 10 mM; ε_[CuL2]483 nm_ = 7840 M^−1^ cm^−1^) [68]. Standard KOH solutions were prepared by diluting carbon dioxide-free concentrated solutions in freshly boiled, doubly distilled water under N_2_ atmosphere. The title of the KOH solution was determined potentiometrically by titrations against potassium hydrogen phthalate. HEPES buffer solution (100 mM, pH 7.4) was prepared by suspending a proper amount of solid HEPES in double distilled water, and then correcting the pH to 7.4 using a concentrated NaOH solution. The buffer solution was passed through a 0.45 μm nylon filter, transferred into an air-free Schlenk vessel, and deoxygenated by purging with a N_2_ stream before storage. The pH was checked every 2–3 days.

### 3.4. Potentiometric Measurements

The potentiometric titrations of R3A peptide and Cu(II)/R3A system were carried out in aqueous solution at *T* = 25.0 ± 0.1 °C and *I* = 0.1 M (KCl) under N_2_ stream, using 1.5 to 1.8 mL sample volumes. The potentiometric apparatus was previously described [68]. A Metrohm semimicro electrode was used, which was calibrated in terms of [H^+^] by titrating HCl solutions with a KOH standardized solution. The electrode parameters were determined using the Gran’s method, [69] and the p*K*_w_ value resulted to be 13.76(1). Protonation studies of R3A peptide were carried out by alkalimetric titrations in the pH range 3.1–10.5 (5 samples; C_peptide_ = 0.56–0.65 mM). Cu^2+^ complex formation studies were carried out by alkalimetric titrations of 3 samples in the pH range 3.1–10.1 (Cu:peptide = 1:1.2–2.1, C_Cu_ = 0.25–0.43 mM). The protonation and complex formation constants of the systems were calculated with the software HyperQuad 2013 [70] and the results were used to draw the species distribution curves with the software Hyss2009 [71].

### 3.5. UV-Visible Absorption and Circular Dichroism Studies

For the Cu(II)/R3A system, visible spectra were collected in the pH range 4.3–10.5 (Cu:peptide = 1:2.1, C_Cu_ = 0.7 mM, 0.1 M KCl). Visible circular dichroism spectra were collected in the pH range 4.3–10.0 (Cu:peptide = 1:2.1, C_Cu_ = 0.7 mM, 0.1 M KCl). All spectra were collected at 25.0 °C with an ionic strength of 0.1 M (KCl). Spectra were collected using quartz cuvettes of 1 cm path length.

For the Cu(I)/R3A and R3C systems, spectrophotometric titrations were carried out in aqueous HEPES buffer solution (100 mM, pH 7.4). All sample solutions were prepared using freshly N_2_-fluxed (at least 5 min) buffer *medium*, which was transferred into the cuvette and added with solid sodium ascorbate (to obtain a 10 mM concentration) prior to reactant mixing. Titrand sample solutions of [Cu(CH_3_CN)_4_]BF_4_ in the presence of 2.15 eq. of Fz^2−^ were prepared directly in the quartz cuvette: 660 µL of nitrogen bubbled buffer solution were added with ca. 1.6 mg of sodium ascorbate to obtain a 10 mM ascorbate concentration. The stock solution of [Cu(CH_3_CN)_4_]BF_4_ (44 μL) was added with 100 µL of the Fz^2−^ solution to obtain final formal concentrations of 0.63 µM and 88 µM for [Cu(CH_3_CN)_4_]BF_4_ and Fz^2−^, respectively. The solution was titrated with R1 or R3 up to Cu:peptide = 1:3. In all samples containing [Cu(CH_3_CN)_4_]BF_4_, the amount of acetonitrile did not exceed 7% total volume. All titrations were performed in triplicate. The spectral absorption data were treated using the software HypSpec2014 [70].

### 3.6. Kinetics of Oxidation of Catechols and Ascorbate in the Presence of [Cu-R3A] and [Cu-R3C] Complexes

The kinetic profiles of dopamine and 4-methylcatechol oxidation by Cu^II^ were studied at 25 °C in 50 mM HEPES buffer at pH 7.4, saturated with atmospheric air. In order to monitor the reaction, the dopaminochrome band at 475 nm for dopamine and the quinone band at 401 nm for 4-methylcatechol were followed. The substrate (3 mM) autoxidation reaction (i.e., the not-catalyzed catechol oxidation by molecular oxygen dissolved in the buffer) was also evaluated. All experiments were performed through the addition of copper(II) nitrate (25 µM) to the catechol substrate (3 mM) and 1 or 2 equiv. of R3A and R3C peptides (25–50 µM). For the kinetic profiles in the presence of ascorbate as substrate, the concentration of the reducing agent was decreased to 0.15 mM and the substrate consumption was followed at 265 nm. To study the catalytic behavior of Cu^I^ complexes with tau fragments, the addition of copper(II) nitrate was substituted with the addition of tetrakis(acetonitrile)copper(I) hexafluorophosphate previously dissolved in argon purged acetonitrile. The solution containing copper(I) and R3 peptides was previously prepared in anaerobic conditions and purged with argon; before starting the acquisition, the substrate was added to the solution and the mixture was then exposed to atmospheric dioxygen. All measurements were performed at least in duplicate.

### 3.7. Kinetics of Oxidation of Catechols in the Presence of Cu and N-Acetylcysteine/Cystine 

The kinetic profiles of dopamine oxidation by Cu^II^ were also studied in the presence of several concentrations of *N*-acetylcysteine or *N*-acetylcystine (0–50 μM) (Scheme S1) in the same conditions previously described. *N*-acetylcystine was synthesized starting from *N*-acetylcysteine (250 mg) dissolved in diluted acetic acid (5 mL) and the generation of the disulfide bond was induced by adding ammonium carbonate to pH 6 and DMSO (10% *v*/*v*) [72]. After 24 h, the product was purified by HPLC and characterized by UV-vis (Abs 260 nm), ^1^H-NMR, and ESI-MS (*m*/*z* 325 [M+H]^+^).

### 3.8. Identification and Characterization of Modified Peptides by HPLC-ESI/MS

The competitive peptide modification during catalysis was studied by analyzing through HPLC-ESI/MS the reaction mixture at different reaction times (10, 30, 60, 90 min). All samples were prepared maintaining the same conditions of the kinetic studies: MC or DA (3 mM), copper(II) nitrate (25 µM), R3A or R3C (25 µM) in 50 mM HEPES buffer at pH 7.4. The elution of both fragments was carried out by using 0.1% HCOOH in distilled water (solvent A) and 0.1% HCOOH in acetonitrile (solvent B), with a flow rate of 0.2 mL/min; elution started with 98% solvent A for 5 min followed by a linear gradient from 98 to 55% A in 65 min.

## 4. Conclusions

The level of copper in Alzheimer’s disease is still controversial as both copper deficiency and copper overload were observed when analyzing samples from different brain regions. Taken together, it seems that an aberrant copper homeostasis could be significantly contributing to Alzheimer’s disease pathology, affecting the aggregation process of neuronal protein, or promoting the general increase in oxidative stress. Despite the emerging evidence of tau protein involvement in Alzheimer’s disease progression, [73], the interaction between copper and tau is poorly characterized, if compared to that with other neuronal proteins, such as α-synuclein or amyloid-β. 

Given the complexity of tau protein, which has considerable size and various potential binding sites for metals, we have chosen a bottom-up approach to fully characterize simpler systems and then increase the degree of complexity. For this reason, in our previous study [47], we have characterized the interaction between copper and R1 peptide (which has only one histidine, as a potential attachment site) and a reduced version of R3 fragment. In particular, Cys322 was excluded to allow a complete characterization of the effect of the neighboring His-His tandem in copper binding. In the present study, we extended the analysis to the whole 18 amino acid peptide R3, including Cys, and to the mutant in which cysteine is replaced by an alanine. The role of Cys322, together with that of Cys291 in R2, is still an object of debate, since the formation of inter- or intra-molecular disulfide bonds seems to increase or decrease, respectively, the aggregation tendency of tau. However, the recently solved structures of tau filaments revealed that the two cysteine residues are not involved in disulfide bond formation and are structurally different since Cys322 is incorporated into the core of the fibril, whereas Cys291 projects away from the core [12].

The present study shows that the presence of cysteine affects both the binding and reactivity of copper bound to R3 fragment of tau protein. Spectrophotometric measurements show that cysteine increases the binding affinity for copper(I) with respect to the R3A mutant or to the “short” R3 fragment used in the previous study. On the other hand, the high reactivity of copper(II)-cysteine system did not allow to evaluate the stability constant for cupric ion, making not possible a comparison with the stability constants obtained with the R3A mutant or with to the “short” R3, or with other important neuronal peptides. 

In addition, the oxidative reactivity of copper complexes with R3C and R3A is different. Copper-R3A confirms that the vicinal His-His tandem forms a coordination sphere that guarantees an efficient copper(I/II) redox cycling, which is testified by a faster oxidation of catechol substrates such as DA and MC, compared to that of free copper. On the other hand, the copper–R3C complex shows a different kinetic behavior in DA oxidation, indicating a mechanism that involves the cysteine residue. In particular, cysteine reacts with intermediate products of DA oxidation, i.e., dopamine semiquinone or DAQ. The first reaction is a radical process, which can lead to the formation of Cys–Cys dimers or to the propagation of radical species, whereas the second generates the formation of a covalent bond between cysteine and dopamine. The HPLC-MS analysis of the reaction mixtures indicates that R3C is extremely sensitive to oxidative modifications and Cys-DA or Cys-DAQ adducts are the preferential products for this reaction, whereas the disulfide bond is a minor product. This result is particularly relevant for the aggregation process of tau, because, as stated above, if the two cysteine residues are not involved in disulfide bond in the fibril, they are still available for post-translational modifications, as the dopamination process. 

In conclusion, in anaerobic conditions the cysteine residue stabilizes the copper(I) binding site, whereas in aerobic conditions it contributes to promote oxidative reactions, especially in combination with redox active metal ions like copper, or reductants such as DA or ascorbate. This conclusion is particularly important for the extracellular space where copper is mainly in the oxidized form and tau can be released in both physiological and pathological conditions [74]. The present study suggests that increasing copper levels and related interaction with tau might contribute to the detrimental tau aggregation and overall increase of oxidative stress.

This study, therefore, is a necessary step that opens the way for subsequent analysis on larger peptide fragments containing two cysteine residues and/or more than one binding site for copper, with the aim of approaching the more complex situation of the whole protein.

## Figures and Tables

**Table 1:** Proton dissociation constants (p*K*_a_) of the fully protonated R3A peptide (H_4_L^4+^), and logarithms of stability constants of the copper(II) complexes of R3A in aqueous solution (*T* = 298.2 K, *I* = 0.1 M in KCl). Standard deviations are given in parentheses.

p*K*_a_ (H_4_L^4+^)			Species	log *β*
p*K*_a1_	5.67(1)	N_im_H^+^ His	[CuH_2_L]^4+^	25.79(1)
p*K*_a2_	6.56(1)	N_im_H^+^ His	[CuHL]^3+^	19.42(2)
p*K*_a3_	9.83(1)	NH_3_^+^ Lys	[CuL]^2+^	12.68(2)
p*K*_a4_	10.50(1)	NH_3_^+^ Lys	[CuLH_−1_]^+^	4.09(3)
			[CuLH_−2_]	−5.84(3)
			[CuLH_−3_]^−^	−16.40(4)

**Table 2:** Percent modification with time of R3A (25 µM) in the presence of copper (25 µM) and DA (3 mM).

Time	R3A	Oxidation		Dopamination		Oxidation and Dopamination	
		+16	+32	+149	+151	+149+16	+151+16
10′	**97**	2	-	1	-	-	-
30′	**92**	5	1	1	-	1	-
60′	**83**	14	3	1	-	-	-
90′	**63**	27	6	1	-	-	-

**Table 3:** Percent modification with time of R3C (25 µM) in the presence of copper (25 µM) and DA (3 mM).

Time	R3C	Oxidation			Dopamination				Oxidation and Dopamination		Dimerization
		+16	+32	+48	+149	+151	+149 +149	+151 +151	+149 +16	+151 +16	
5′	**29**	1	1	1	9	52	1	1	1	2	2
10′	**5**	1	1	1	13	65	2	1	4	4	4
30′	**2**	-	1	-	12	70	2	2	3	3	4

**Table 4:** Percent modification with time of R3C (25 µM) in the presence of copper (25 µM), DA (3 mM) and *N*-acetylcysteine (25 µM).

Time	R3C	Oxidation			Dopamination				Oxidation and Dopamination		Dimerization	-AcCys Addition
		**+16**	**+32**	**+48**	**+149**	**+151**	**+149** **+149**	**+151** **+151**	**+149** **+16**	**+151** **+16**		
10′	**42**	1	1	1	5	34	1	1	2	1	2	9

## Supplementary Materials

The supporting information can be downloaded at: https://www.mdpi.com/article/10.3390/ijms231810726/s1.
