# Supplementary material for: Role of the Cysteine in R3 Tau Peptide in Copper Binding and Reactivity
Source: Int J Mol Sci. 2022 Sep 14;23(18):10726. doi: 10.3390/ijms231810726 (PMC9503722; doi:10.3390/ijms231810726)
Supplement: Supplementary file 1 [file ijms-23-10726-s001.zip › ijms-1860215-supplementary.pdf]

Supporting information

Role of the Cysteine in R3 tau Peptide in Copper Binding and Reactivity

Chiara Bacchella [1], Silvia Gentili [2], Sara Ida Mozzi [1], Enrico Monzani [1], Luigi Casella [1], Matteo Tegoni [2] and Simone Dell'Acqua [1]

[1] Dipartimento di Chimica, Università di Pavia, Via Taramelli 12, 27100 Pavia, Italy
[2] Dipartimento di Scienze Chimiche, della Vita e della Sostenibilità Ambientale, Università di Parma, Parco Area delle Scienze 11/A, 43124 Parma, Italy

Description of the speciation of the system Cu(II)/R3A at pH above 7.6

Moving from pH 7.6 to 9.4 the absorption maximum shifts to 525 nm with the appearance, at pH 8.5, of two overlapping absorption bands. These spectral data are consistent with a (N_{im}, 3x N^-) coordination mode of the $[Cu(LH_{-1})]^+$ species for which a λ_{max} of 523 nm is expected (Scheme 1c). Importantly, no shift of the absorption maximum and no significant change in the pattern of the CD spectra were observed from pH 9.4 to 10.5 (Figure 3b) This suggests that the coordination sphere of Cu^{2+} is the same in each of the species that are predominant above pH 8.6. Hence, it can be stated that the two deprotonation processes leading from $[Cu(LH_{-1})]^+$ to $[CuLH_{-3}]^-$ mainly involve only the two Lys residues which remain non-coordinated. Overall, these potentiometric and spectrophotometric analyses demonstrated that the R3 and R3A peptides behave in the same way in terms of copper(II) binding. Conversely, no information using these techniques could be obtained for the R3C peptide since attainment of equilibrium conditions was not possible in the presence of Cu(II) even at low pH values, likely due to the oxidation of the thiol groups promoted by Cu(II).

Details on the procedure for the data treatment for the calculation of the conditional formation constants of Cu(I)/peptide species

The calculations were carried out using the HypSpec software (Gans, P.; Sabatini, A.; Vacca, A. Investigation of equilibria in solution. Determination of equilibrium constants with the HYPERQUAD suite of programs. *Talanta* **1996**, *43*, 1739-1753). The software requires as input data the spectra dataset, the analytical concentrations of the components (Cu(I), indicator and peptide) and the formation constants of the species, including trial values for the K_f (K_f=1/K_d) that need to be refined during data processing. Also, for species that absorb in the treated spectral range, the molar spectra can be either introduced as input data if known, or calculated as a fitting parameter set. Taking into account that the $[CuL_2]$ species is the only absorbing in the examined spectral range (L = ferrozine or BCA) and that we used cuvettes of 1 cm path length, the equation that describes the absorbance value at a certain wavelength (λ) is:

$$A^{\lambda}_{calc} = \varepsilon^{\lambda}_{calc}[CuL_2] \qquad \text{(S1)}$$

The exchange equilibrium that takes place in solution by addition of the peptide (P) to a solution of [CuL$_2$] (L = indicator) is (charges omitted):

[CuL$_2$] + P = [CuP] + 2 L (S2)

The constant of the exchange equilibrium (K_{exch}) relates to the formation constants of the [CuL$_2$] and [CuP] species ($K_f^{CuL2} = \frac{[CuL_2]}{[Cu][L]^2}$; $K_f^{CuP} = \frac{[CuP]}{[Cu][P]} = \frac{1}{K_d}$) as follows:

$$K_{exch} = \frac{[CuP][L]^2}{[CuL_2][P]} = \frac{K_f^{CuP}}{K_f^{CuL_2}} \qquad (S3)$$

By combining eq. S3 with eq. S1 it results:

$$A_{calc}^{\lambda} = \varepsilon_{calc}^{\lambda} \frac{K_f^{CuL_2}}{K_f^{CuP}} \frac{[CuP][L]^2}{[P]} \qquad (S4)$$

In this equation $K_f^{CuL_2}$ and $\varepsilon_{calc}^{\lambda}$ are known parameters. By least-square regression, in which the sum of squares $\left|A_{obs}^{\lambda} - A_{calc}^{\lambda}\right|^2$ is minimized, K_f^{CuP} is the only fitting parameter. The concentrations of the free species [CuP], [L] and [P] are iteratively calculated from mass balance equations during the least-square iteration processes. The least-square regression provides with the standard deviation of K_f^{CuP}.

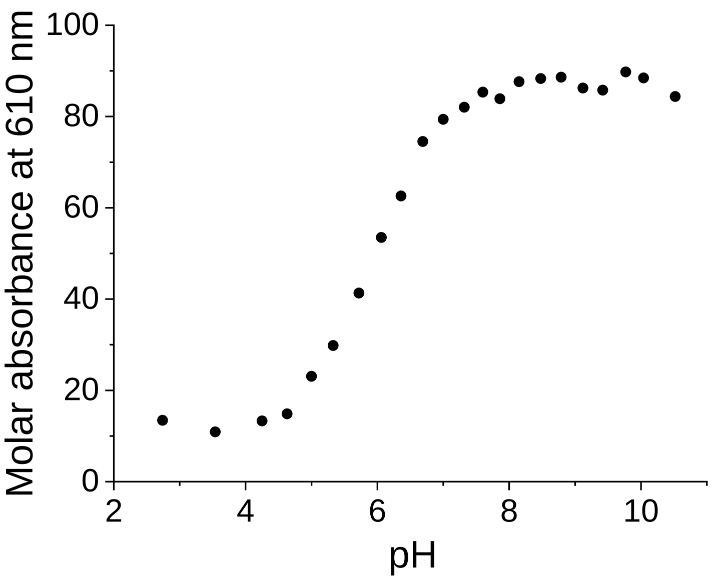

Figure S1. Plot of the molar absorption values at 610 nm (absorbance/Total [Cu]) of solutions of copper(II) and R3A at different pH (Cu^{2+}/R3A = 1:2.1, [Cu] = 0.70 mM, T = 298.2 K, I = 0.1 M in KCl).

Table S1. Spectroscopic absorption features for the system Cu(II) / R3A (L = R3A). Cu/R3A = 1:2.1, [Cu] = 0.55 mM, T = 298.2 K, I = 0.1 M (KCl). See figure 3.

pH	λ_{max}	ε^a	Prevalent species
**5.0**	705	30	$[Cu(H_2L)]^{4+}$
**5.7**	660	46	$[Cu(H_2L)]^{4+}$
**6.4**	620	63	$[Cu(HL)]^{3+}$
**6.7**	606	74	$[Cu(HL)]^{3+}$
**7.6**	585	89	$[CuL]^{2+}$
**8.5**	550	97^b	$[CuL]^{2+}$; $[Cu(LH_{-1})]^{+}$
**9.4**	531	117	$[Cu(LH_{-1})]^{+}$
**10.5**	535	125	$[Cu(LH_{-2})]$

[a] Absorbance/Total [Cu]; [b] Shoulder at 588 nm

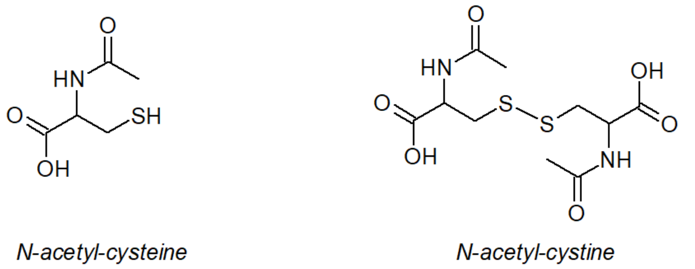

Scheme S1. *N*-acetyl-cysteine and *N*-acetyl-cystine structures.

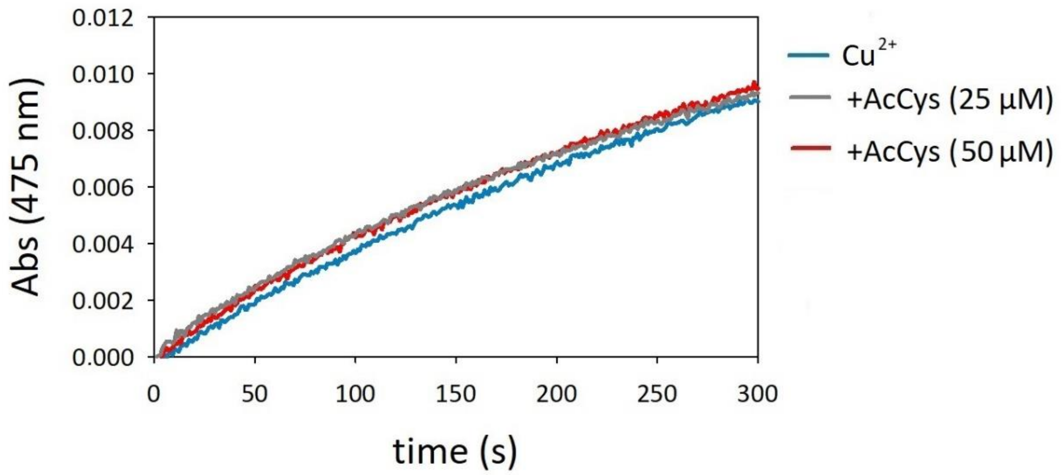

Figure S2. Kinetic profiles of DA (3 mM) oxidation with time in 50 mM HEPES buffer at pH 7.4 and 25°C catalyzed by copper(II) alone (25 µM, light blue trace) and in the presence of *N*-acetyl-cystine (25 µM, grey and 50 µM, red).

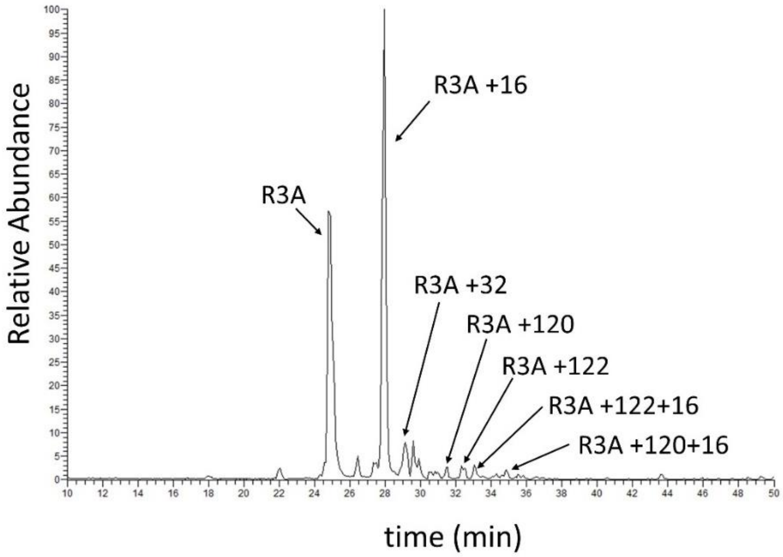

Figure S3. HPLC-MS elution profiles of R3A peptide (25 μM) in 50 mM HEPES buffer at pH 7.4 in the presence of copper(II) (25 μM) and MC (3 mM) after 10 min reaction time. The modifications shown in the chromatogram are the insertion of single (+16)/ double (+32) *O*-atoms, the covalent addition of Cat (+122) and +Q (+120) and the mixed modification of oxygen insertion and Cat/Q adduct formation.

Table S2. Percent modification with time of R3A peptide (25 μM) in the presence of copper (25 μM) and MC (3 mM).

time	R3A	Oxidation		-Cat/Q Addition		Oxidation and -Cat/Q Addition	
		+16	+32	+120	+122	+120+16	+122+16
10	**48**	29	10	3	2	3	5
30	**11**	51	18	1	2	7	10
60	**7**	52	18	2	2	9	10
90	**5**	53	11	3	2	8	18

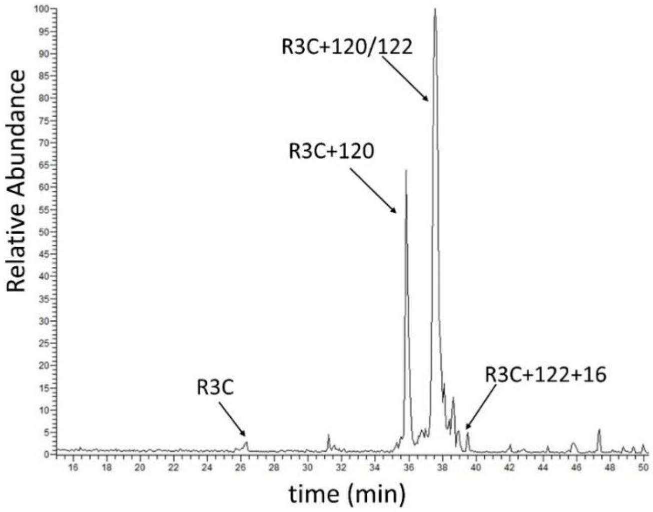

Figure S4. HPLC-MS elution profiles of R3C peptide (25 μM) in 50 mM HEPES buffer at pH 7.4 in the presence of copper(II) (25 μM) and MC (3 mM) after 10 min reaction time. The modifications shown in the

chromatogram are the covalent addition of Cat (+122) and +Q (+120) and the mixed modification of *O*-atom insertion and Cat-adduct formation.

Table S3. Percent modification with time of R3C peptide (25 μM) in the presence of copper (25 μM) and MC (3 mM).

time	R3C	Oxidation		-Cat/Q Addition				Oxidation and -Cat/Q Addition		Dimerization
		+16	+32	+120	+122	+120 +120	+122 +122	+120 +16	+122 +16	
5′	**2**	-	-	43	47	1	2	2	2	1
10′	**-**	1	-	42	42	2	7	3	3	1

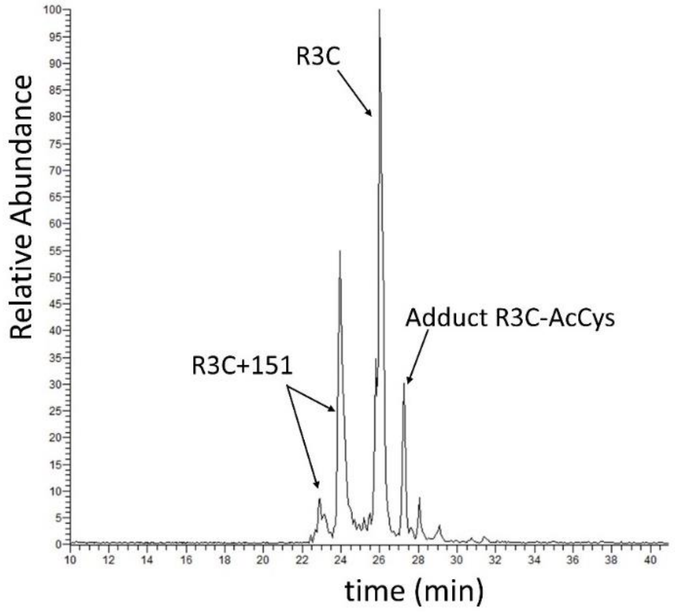

Figure S5. HPLC-MS elution profiles of R3C peptide (25 μM) in 50 mM HEPES buffer at pH 7.4 in the presence of copper(II) (25 μM), DA (3 mM) and *N*-acetylcysteine (25 μM) after 10 min reaction time. The modifications shown in the chromatogram are the covalent addition of DA (+151) or of Ac-Cys (R3C-AcCys).
